# The Role of Human Epididymis Protein 4 in the Diagnosis and Prognosis of Diseases: An Umbrella Review of Systematic Reviews and Meta-Analyses of Observational Studies

**DOI:** 10.3389/fmed.2022.842002

**Published:** 2022-03-24

**Authors:** Ming-Li Sun, Zhi-Yong Yang, Qi-Jun Wu, Yi-Zi Li, Xin-Yu Li, Fang-Hua Liu, Yi-Fan Wei, Zhao-Yan Wen, Bei Lin, Ting-Ting Gong

**Affiliations:** ^1^Department of Obstetrics and Gynecology, Shengjing Hospital of China Medical University, Shenyang, China; ^2^Department of Cardiology, Shengjing Hospital of China Medical University, Shenyang, China; ^3^Clinical Research Center, Shengjing Hospital of China Medical University, Shenyang, China; ^4^Department of Clinical Epidemiology, Shengjing Hospital of China Medical University, Shenyang, China

**Keywords:** diagnosis, GRADE, human epididymis protein 4, prognosis, umbrella review

## Abstract

**Background:**

The application of human epididymis protein 4 (HE4) in diverse health diseases, especially in cancers, has been extensively studied in recent decades. To summarize the existing evidence of the aforementioned topic, we conducted an umbrella review to systematically evaluate the reliability and strength of evidence regarding the role of HE4 in the diagnostic and prognostic estimate of diverse diseases.

**Methods:**

Electronic searches in PubMed, Web of Science, and Embase databases were conducted from inception to September 16, 2021, for meta-analyses, which focus on the role of HE4 in the diagnosis and prognosis of diseases. This study protocol has been registered at PROSPERO (CRD42021284737). We collected the meta-analysis effect size of sensitivity, specificity, positive predictive value, and negative predictive value from diagnostic studies and gathered the hazard ratio (*HR*) of disease-free survival, overall survival, and progression-free survival from prognostic studies. For each systematic review and meta-analysis, we used a measurable tool for evaluating systematic reviews and meta-analysis (AMSTAR) to evaluate the methodological quality. Additionally, we assessed the quality of evidence on estimating the ability of HE4 in the diagnosis and prognosis of diverse diseases by the Grading of Recommendations Assessment, Development and Evaluation (GRADE) guideline.

**Results:**

Overall, 20 meta-analyses including a total of 331 primary studies of different diseases were examined, mainly including ovarian cancer (OC) (*n* = 9), endometrial cancer (EC) (*n* = 6), and lung cancer (LC) (*n* = 4). The methodological qualities of all studies were rated as moderate (45%) or high (55%) by the AMSTAR. According to the GRADE, the certainties of 18 diagnostic pieces of evidence (9 for sensitivity and 9 for specificity) were rated as moderate (34%), low (33%), and very low (33%). Moreover, outcomes from prognosis studies showed evidence (1 for disease-free survival) with high certainty in regard to cancers (such as EC, OC, and LC) with the remaining three being moderate.

**Conclusion:**

This umbrella review suggested that HE4 was a favored biomarker in the prognosis of cancers, which was supported by high certainty of evidence. Additionally, HE4 could provide a suitable method for the diagnosis of EC, OC, and LC with moderate certainty evidence. Further large prospective cohort studies are needed to better elucidate the diagnostic and prognostic role of HE4 in diseases.

## Introduction

The existence or quantitative change of biomarkers can indicate the nature of the diseases and contribute to a clear understanding of the progress of the diseases (1). Biomarker-disease relationships have been extensively investigated. Compared with other diagnostic methods, serum biomarkers, such as carcinoembryonic antigen (2) and squamous cell carcinoma antigen (3) take priority due to their low inspection cost and non-invasiveness, which is an essential step for the screening, diagnosis, classification, and prognosis of diverse diseases. Carbohydrate antigen 125 (CA125) levels are widely used for the diagnosis and prognosis of diseases (4, 5), but human epididymis protein 4 (HE4) has a higher true positive rate and true negative rate than CA125, so the clinical application of HE4 is wider than that of CA125. HE4 is a secretory glycoprotein, transcribed and translated from the whey acidic protein 4-disulfide core domain 2 genes (6). HE4 was known as one of the most promising novel serum biomarkers for the diagnosis, prognosis, and monitoring of diverse diseases, and its ability on disease diagnosis was approved and supported by Food and Drug Administration (7, 8). HE4 is considered as a potential biomarker for ovarian cancer (OC) because it shows higher specificity and diagnostic accuracy than other biomarkers (9). The functions of HE4 in the field of biomarkers were found in 2003 (10). Considering the two well-researched protein co-expression genes near HE4, secretory leukocyte protease inhibitor and P13, which are demonstrated to have angiogenesis regulation, cell growth, cell migration, immune, antimicrobial, and anti-HIV functions (11, 12).

Previous epidemiological studies have investigated the diagnostic role of HE4 in some diseases, such as OC (13, 14), lung cancer (LC) (15), renal fibrosis (16), breast cancer (17), and endometrial carcinoma (EC) (18). For example, the results of a meta-analysis showed that HE4 had higher specificity (0.84 vs. 0.57) and similar sensitivity (0.79 vs. 0.81) than CA125 for differentiating malignant from benign pelvic mass disease (19), similar findings could be observed in those meta-analyses (7, 20, 21). However, a meta-analysis demonstrated that HE4 was no better than CA125 for OC prediction (22). Due to the high disease heterogeneity and risk of bias, there is no consensus on the accuracy of HE4 in different diseases. On the other hand, the prognostic role of HE4 expression in diseases has also been reported (23–26). However, the findings from these studies remain controversial. For example, Kalapotharakos et al. (23) reported that the serum level of HE4 was a prognostic marker for overall survival (OS) among Sweden women with epithelial OC. In addition, a previous study (25) reported that 2-year OS was not associated with HE4 levels (positive vs. negative). The disparity in these studies is probably attributed to different kinds of diseases, histological types of the same disease, the proportion of the usual population, and methods/instruments for the measurement of HE4 levels. Therefore, we sought a method to evaluate the diagnostic value (sensitivity, specificity, positive likelihood ratio, negative likelihood ratio, and diagnostic odds ratio) and prognostic value [OS, disease-free survival (DFS), and progression-free survival (PFS)] of HE4 in diverse diseases.

Umbrella reviews could find the disparity, analyze the reason, and summarize the information of a specific topic. In the umbrella review, the strength and credibility of associations can be assessed using standardized methods, such as evaluating bias or grading the evidence (27). Umbrella reviews can provide an overall examination of the role of HE4 in the diagnosis and prognosis of diseases, and compare and contrast the results of published systematic reviews. Hence, we conducted an umbrella review to consolidate the existing evidence to estimate the ability of HE4 in the diagnosis and prognosis of diverse diseases.

## Methods

### Literature Search and Eligibility Criteria

We performed an umbrella review, which was the systematic collection and assessment of multiple systematic reviews and meta-analyses about the role of HE4 in the diagnosis and prognosis of diseases. The report of this umbrella review followed the recommendations of the Preferred Reporting Items for Systematic Reviews and Meta-Analyses (PRISMA) group (28). Our protocol has been registered in PROSPERO (CRD42021284737). PubMed, Web of Science, and Embase databases were searched from inception to September 16, 2021, for related systematic reviews and meta-analyses. In addition, we hand-searched the reference lists of eligible articles to prevent omissions. The present search strategy used the following keywords: “(human epididymis 4 OR HE4 protein OR human epididymis secretory protein 4 OR wap 4-disulfide core domain proteins 2 OR whey acidic protein four disulfide core protein 2) AND (meta-analysis OR systematic overview OR systematic review)” (Supplementary Appendix 1).

### Inclusion and Exclusion Criteria

Two authors (Sun ML and Li YZ) screened all records independently. Differences were resolved through consensus with the third author (Gong TT). The inclusion criteria were as follows: (1) published systematic reviews and the meta-analyses of observational studies in English; (2) articles assessing the role of HE4 in the diagnosis and prognosis of diseases; (3) studies providing critical data [diagnosis (sensitivity, specificity, positive likelihood ratio, negative likelihood ratio, and diagnostic odds ratio) and prognosis (PFS, DFS, OS, disease-specific survival, mortality, and progression/recurrence)]. This topic defined exposure as HE4 and outcome as the diagnosis and prognosis of diverse diseases. When two or more systematic reviews and meta-analyses examined the exact same exposures and outcomes, we included the larger or largest number of original studies to avoid duplicate assessments of the same topic (29).

We excluded articles if they met the following criteria: (1) narrative reviews, systematic reviews, and meta-analyses that involved fewer than three original studies; (2) articles that did not report necessary study-specific data (30–32); (3) studies exploring genetics or experiments in animals, *in vitro*, and *in vivo*; (4) full texts that were not available. For each eligible systematic review and meta-analyses, we collected all the exposure and outcome of the study we were interested in, such as subgroup analysis and dose-response analysis.

### Data Extraction

Relevant data from each included systematic review and meta-analysis were extracted by two investigators (Sun ML and Li YZ) independently. The final decision was reached by a third investigator (Gong TT) when in case of discrepancies. From each eligible systematic review and meta-analysis, we recorded the first author name, publication year, journal, exposure, effect sizes, number of studies, and outcomes. For the information of the original diagnostic study, we further extracted the first author, publication year, cutoff value, case number, total population, and adjusted estimates (sensitivity, specificity, positive likelihood ratio, negative likelihood ratio, and diagnostic odds ratio). For the primary studies on prognosis, we extracted event numbers, total populations, comparisons, and effect size [hazard ratio (*HR*)]. If necessary, we would search for information from primary studies to find the missing data.

### Evaluation of the Quality of Included Meta-Analyses

The quality of each eligible systematic review and meta-analyses was evaluated based on a measurement tool for evaluating systematic reviews and meta-analyses (AMSTAR) (33). The evaluations were performed independently by two researchers (Sun ML and Li YZ) and determined by a third researcher (Gong TT) when differences occurred. The AMSTAR tool is an 11-item questionnaire that asks reviewers to answer yes, no, cannot answer, or not applicable (33). If an item of the criteria is met with “yes,” one point will be awarded. The final total score of 11-items can measure the methodological quality of systematic reviews and meta-analyses. The AMSTAR score was graded as high (8–11), moderate (4–7), and low (0–3) quality (33).

### Grading of the Evidence

We used the Grading of Recommendations Assessment, Development, and Evaluation (GRADE) principles to evaluate the credibility of evidence from the eligible systematic reviews and meta-analyses (34–36). Each body of evidence was evaluated independently by two authors (Sun ML and Li YZ), and the third author (Gong TT) made the decision when differences arose. There were four levels of evidence through the GRADE tool: high, moderate, low, and very-low quality (37–40). For the studies of diagnostic test accuracy, a body of evidence begins with high certainty (34). Moreover, there was no sufficient guidance available on the application of the three GRADE criteria for upgrading evidence related to diagnostic test accuracy studies (35), thus we downgraded the evidence on the basis of prespecified criteria. The GRADE method proposes 5 factors rating down certainty in the evidence, such as the risk of bias, inconsistency, indirectness, imprecision, and publication bias (35, 41). Besides, for prognostic studies, the initial quality of evidence was high. We downgraded the quality of the evidence (risk of bias, inconsistency, indirectness, imprecision, and publication bias) and upgraded the quality (large effect, dose response, and plausible residual confounding) (41).

## Results

### Study Selection

**FIGURE 1:**
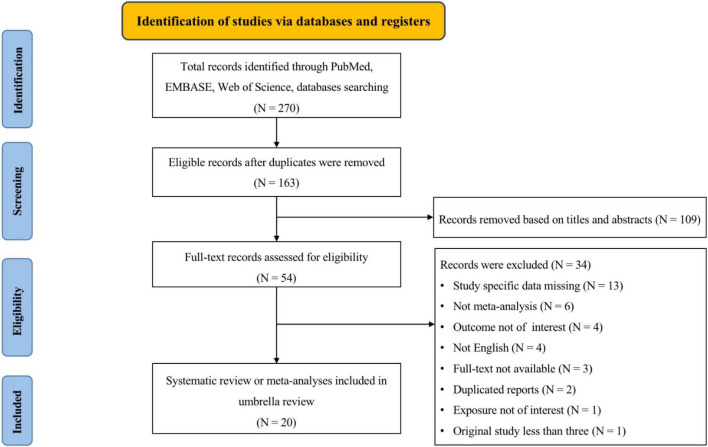
A flowchart of a selection of studies for inclusion in an umbrella review on human epididymis protein 4 (HE4) and the diagnosis and prognosis of diseases.

Overall, the search retrieved 270 articles from three databases (Figure 1). After the removal of duplicates, 163 articles were identified. Then after screening the titles and abstracts, 109 articles were excluded. Subsequently, a further 34 articles were excluded for the following reasons: 13 presented insufficient data, 6 did not conduct meta-analysis, 4 explored outcomes that we were not interested in, 4 published not in English, the full text of 3 articles were not available, 2 published duplicated reports, 1 explored exposure that we were not interested in, and 1 conducted less than three original studies. Ultimately, 20 studies (7, 8, 15, 19–22, 42–54) were eligible to be included in the main analysis.

### Diagnostic Meta-Analysis

As reported in Table 1, 250 primary studies of 17 meta-analyses were included in the 16 articles for diagnosis (7, 15, 19–22, 42, 43, 45–50, 52, 53). These sixteen articles were published between 2012 and 2021. The studies enrolled in the meta-analyses were all diagnostic accuracy tests, and the median number of original studies per meta-analysis was 12 (range from 4 to 45). The number of participants ranged from 891 to 10,671, and the number of cases exceeded 1,000 in 10 meta-analyses. The median cutoff value of HE4 ranged from 19 to 82 pmol/L. HE4 had the highest diagnostic value in OC (48) with an area under the curve (AUC) of 0.96 [95% confidence interval (*CI*) = 0.95–0.98], and the lowest diagnostic value in EC (46) with an AUC of 0.75 (95% *CI* = 0.81–0.87). The numbers of meta-analysis evaluating the role of HE4 expression in the diagnosis of OC, EC, LC, EOC, and malignant pelvic mass were seven, five, three, one, and one, respectively. Furthermore, twelve and five meta-analyses systematically evaluated the quality of the included studies according to the Quality Assessment of Diagnostic Studies version 2 (QUADAS-2) tool and the Quality Assessment of Diagnostic Accuracy Studies (QUADAS) tool, respectively.

**TABLE 1:** Main characteristics of the included systematic reviews or meta-analysis that evaluate the role of HE4 in the diagnosis of diseases.

Outcome	References	effect size	No. of studies	No. of population	No. of cases	Cut-off (pmol/L), MD	AUC	Quality of assessment
Endometrial cancer	Liu et al. (46)	SE, SP, PLR, NLR, DOR	17	3,167	1,807	74	0.75	QUADAS-2
	Chen et al. (43)	SE, SP, DOR	8	1,832	1,129	70	0.77	QUADAS
	Bie et al. (49)	SE, SP, DOR	6	1,551	791	64	0.83	QUADAS-2
	Hu et al. (7)	SE, SP, DOR	21	4,623	2,229	75	0.78	QUADAS-2
	Li et al. (21)	SE, SP	12	3,150	1,442	77	0.88	QUADAS
Ovarian cancer	Jia et al. (52)	SE, SP, PLR, NLR, DOR	7	986	413	19	0.93	QUADAS-2
	Ferraro et al. (20)	SE, SP, PLR, NLR	13	3,471	1,200	74	N/A	QUADAS-2
	Yu et al. (47)	SE, SP, PLR, NLR	12	2,607	779	72	0.95	QUADAS
	Yang et al. (48)	SE, SP, PLR, NLR	31	7,045	2,112	70	0.96	QUADAS-2
	Huang et al. (53)	SE, SP	18	4,673	1,369	72	0.91	QUADAS-2
	Macedo et al. (50)	SE, SP	45	10,671	3,946	74	0.92	QUADAS
	Li et al. (22)	SE, SP	5	891	388	74*^a^*	0.95	QUADAS-2
Epithelial ovarian cancer	Li et al. (22)	SE, SP	4	916	265	70*^a^*	0.95	QUADAS-2
Malignant pelvic mass	Olsen et al. (19)	SE, SP	7	1,403	433	72	N/A	QUADAS-2
Lung cancer	Cheng et al. (42)	SE, SP, PLR, NLR, DOR	7	1,245	715	75	0.86	QUADAS
	He et al. (15)	SE, SP, PLR, NLR	21	3,599	1,893	82	0.86	QUADAS-2
	Yan et al. (45)	SE, SP	16	3,202	1,756	77	0.86	QUADAS-2

*DOR, diagnostic odds ratio; HE4, human epididymis protein; MD, median; NLR negative likelihood ratio; N/A, not available; PLR, positive likelihood ratio; QUADAS, Quality Assessment of Diagnostic Studies tool; QUADAS-2, Quality Assessment of Diagnostic Accuracy Studies version 2 tool; SE, sensitivity; SP, specificity.*

*^a^The unit is PM in the systematic review conducted by Li et al. (22).*

### Prognostic Meta-Analysis

Table 2 showed the nine meta-analyses that involved 81 original studies in four articles for prognosis (8, 44, 51, 54). These 4 articles were published between 2017 and 2020. Each meta-analysis combined 3–23 original cohort study estimates, with a median of 8. The number of participants ranged from 484 to 3,564. The lowest number of events in the meta-analyses was 63 and the highest number was 1,051. The types of outcomes in these meta-analyses included EC, OC, LC, and gastric cancer (GC). Of the nine meta-analyses, four, two, and three meta-analyses assessed the association between HE4 expression and OS, DFS, and PFS, respectively. Among nine meta-analyses, only four conducted the quality assessment for individual studies by the Newcastle-Ottawa Quality Assessment Scale (NOS).

**TABLE 2:** Main characteristics of the included systematic reviews or meta-analysis that evaluate the role of HE4 in the prognosis of diseases.

Outcomes	References	Disease type	Level of comparison	Effect size, HR	No. of studies	No. of population	No. of events	Quality of assessment
Overall survival	He et al. (54)	EC	High vs. low	2.71 (1.28, 4.15)	5	755	206	N/A
	Dai et al. (44)	EC, OC, LC, GC		2.15 (1.77, 2.62)	23	3,564	1,051	NOS
	Yuan et al. (51)	OC		1.91 (1.40, 2.61)	9	1,152	367	N/A
	Zhong et al. (8)	LC		1.73 (1.19, 2.52)	7	1,312	378	NOS
Disease-free survival	He et al. (54)	EC		2.00 (1.16, 2.84)	5	971	94	N/A
	Dai et al. (44)	EC, OC, LC, GC		2.50 (1.86, 3.37)	8	1,307	133	NOS
Progression-free survival	He et al. (54)	EC		2.81 (0.72, 4.90)	3	484	63	N/A
	Dai et al. (44)	EC, OC, LC, GC		1.27 (1.11, 1.45)	10	1,272	389	NOS
	Yuan et al. (51)	OC		1.38 (1.13, 1.69)	11	1,102	462	N/A

*EC, endometrial cancer; GC, gastric cancer; HE4, human epididymis protein; HR, hazard ratio; LC, lung cancer; MOOSE, Meta-analysis of Observational Studies in Epidemiology guidelines; NOS, Newcastle-Ottawa Quality Assessment Scale; N/A, not available; OC, ovarian cancer.*

### The Methodological Quality of the Meta-Analyses

Among the 20 articles (both for diagnosis and prognosis) included in our umbrella review, 11 (55%) articles were rated as high quality and nine (45%) were defined as a moderate quality based on the AMSTAR criteria (Supplementary Figure 1). The common flaws were that gray literature was not considered in the literature search (85%), and the list of excluded studies was not presented (100%).

### Grading of Evidence

In total, eighteen and four pieces of evidence met the conditions of the GRADE, which were from nine diagnostic articles (7, 15, 20, 21, 42, 43, 48, 52, 53) and two prognostic articles (8, 44), respectively. Due to the lack of the quality of assessment and bias publication, eighteen pieces of evidence were eventually evaluated by the GRADE assessment (Table 3). No evidence in diagnosis was rated as high evidence, whereas six of the pieces of evidence were supported by moderate. In addition, six pieces of evidence presented low evidence, and the others were supported by very-low evidence.

**TABLE 3:** The results of GRADE assessment of the evidence certainty on the association between the diagnostic accuracy of HE4 and diverse diseases.

Outcome	References	Outcomes rated	Downgrade factors					Certainty of the evidence
			Risk of bias	Imprecision	Inconsistency	Indirectness	Publication bias	
Endometrial cancer	Chen et al. (43)	SE	No serious	No serious	Serious limitation^b^	Serious limitation^d^	No serious	⊕⊕○○ low
		SP	No serious	No serious	Serious limitation^b^	Serious limitation^d^	No serious	⊕⊕○○ low
	Hu et al. (7)	SE	No serious	No serious	Serious limitation^b^	No serious	Serious limitation^e^	⊕⊕○○ low
		SP	No serious	No serious	Serious limitation^b^	No serious	Serious limitation^e^	⊕⊕○○ low
	Li et al. (21)	SE	No serious	No serious	Serious limitation^b^	No serious	No serious	⊕⊕⊕○ moderate
		SP	No serious	No serious	Serious limitation^b^	No serious	No serious	⊕⊕⊕○ moderate
Ovarian cancer	Jia et al. (52)	SE	Serious limitation^a^	No serious	Serious limitation^b^	No serious	No serious	⊕⊕○○ low
		SP	Serious limitation^a^	No serious	Serious limitation^b^	No serious	No serious	⊕⊕○○ low
	Ferraro et al. (20)	SE	Serious limitation^a^	No serious	Serious limitation^c^	No serious	Serious limitation^e^	⊕○○○ very low
		SP	Serious limitation^a^	No serious	Serious limitation^b^	No serious	Serious limitation^e^	⊕○○○ very low
	Yang et al. (48)	SE	Serious limitation^a^	No serious	Serious limitation^b^	No serious	Serious limitation^e^	⊕○○○ very low
		SP	Serious limitation^a^	No serious	Serious limitation^b^	No serious	Serious limitation^e^	⊕○○○ very low
	Huang et al. (53)	SE	No serious	No serious	Serious limitation^c^	No serious	No serious	⊕⊕⊕○ moderate
		SP	No serious	No serious	Serious limitation^b^	No serious	No serious	⊕⊕⊕○ moderate
Lung cancer	Cheng et al. (42)	SE	No serious	No serious	Serious limitation ^b^	No serious	No serious	⊕⊕⊕○ moderate
		SP	No serious	No serious	Serious limitation^c^	No serious	No serious	⊕⊕⊕○ moderate
	He et al. (15)	SE	Serious limitation^a^	No serious	Serious limitation^b^	Serious limitation^d^	No serious	⊕○○○ very low
		SP	Serious limitation^a^	No serious	Serious limitation^b^	Serious limitation^d^	No serious	⊕○○○ very low

*SE, sensitivity; SP, specificity.*

*^a^Downgraded by one level for the risk of bias: this domain was downgraded by 1 level because the Quality Assessment of Diagnostic Accuracy Studies version 2 tool.*

*^b^Downgraded by only one level for inconsistency: substantial heterogeneity is seen between studies (I^2^ > 75%), whereas heterogeneity was mainly explained.*

*^c^Downgraded by one level for inconsistency: substantial heterogeneity is seen between studies (50% < I^2^ < = 75%).*

*^d^Downgraded by one level for indirectness: the concentration of HE4 measured by different test methods.*

*^e^Downgraded by one level for publication bias: asymmetry on funnel plot, the value of p of Deek’s test < 0.1.*

Only four pieces of evidence were graded by GRADE in the umbrella review, and only one association between HE4 expression and DFS was supported by high evidence (Table 4). Moreover, the association between HE4 and PFS/OS presented moderate evidence (*n* = 3). There was no association indicating low or very-low evidence.

**TABLE 4:** The results of GRADE assessment of the evidence certainty on the association between HE4 and diverse diseases in prognosis.

Outcome	References	Downgrade factors					Upgrade factors			Certainty of the evidence
		Risk of bias	Imprecision	Inconsistency	Indirectness	Publication bias	Large effect	Dose-response	Plausible confounding	
OS^†^	Dai et al. (44)	Not serious	Not serious	Serious limitation^a^	Not serious	Serious limitation^c^	Yes^d^	No	Would Not Reduce Effect	⊕⊕⊕○ moderate
OS^‡^	Zhong et al. (8)	Not serious	Not serious	Serious limitation^b^	Not serious	Not serious	No	No	Would Not Reduce Effect	⊕⊕⊕○ moderate
DFS*	Dai et al. (44)	Not serious	Not serious	Not serious	Not serious	Not serious	Yes^d^	No	Would Not Reduce Effect	⊕⊕⊕⊕ high
PFS*	Dai et al. (44)	Not serious	Not serious	Not serious	Not serious	Serious limitation^c^	No	No	Would Not Reduce Effect	⊕⊕⊕○ moderate

*DFS, disease-free survival; OS, overall survival; PFS, progression-free survival.*

*^†^Prognosis of cancers, such as EC, OC, LC, and GC.*

*Prognosis of cancers, such as EC, OC, and LC. ^‡^Overall survival of LC.

*^a^Downgraded by one level for inconsistency: substantial heterogeneity is seen between studies (I^2^ > 50%).*

*^b^Downgraded by only one level for inconsistency: substantial heterogeneity is seen between studies (I^2^ > 75%), whereas heterogeneity was mainly explained.*

*^c^Downgraded by one level for publication bias: asymmetry on funnel plot, the p of Egger’s test or Begg’s test < 0.05.*

*^d^Upgraded by only one level for large effect: because of the large magnitude of effect (HR > 2.0).*

## Discussion

### Principal Findings

In this umbrella review, we provided an overview of the role of HE4 in the diagnosis and prognosis of diverse diseases using the AMSTAR tool and GRADE guidelines. In the study exploring the diagnostic role of HE4, six associations (outcomes of OC, EC, and LC) were supported by moderate evidence, and all of them had serious limitations of inconsistency in the evaluation by GRADE guideline. For the prognostic studies, there was only one evidence of HE4 performing its action in the clinical setting as a suitable biomarker with high certainty while the remaining three associations were moderate. The key findings echo the scientific question we raised at the outset of the present study, whether HE4 could be a useful biomarker for disease diagnosis and prognosis.

### Explanation Findings From Prognostic Meta-Analyses

According to the criteria of the GRADE guideline, there was one high-quality evidence, Dai et al. (44) conducted a meta-analysis and reported that the HE4 level can provide a useful prognostic biomarker (only for DFS) for patients with cancers (987 patients with EC, 211 patients with LC, and 98 patients with OC). The prognostic effect of HE4 on the disease was mainly focused on OC, but a systematic review suggested that the prognostic role of HE4 in other types of cancers needed more endeavor than before (55). Previous articles or meta-analyses suggested that OC (23, 56), EC (57), LC (58), and GC (59) patients with a high concentration of HE4 have shorter survival or more likely metastasis than those with low concentrations. However, to the best of our knowledge, our umbrella review is the first to present the evidence from previous systematic reviews and meta-analyses regarding the roles of HE4 in the diagnosis and prognosis of diverse diseases. Besides, we found that the prognosis had a higher quality level of evidence than the diagnosis. Of note, the risk of bias was the main reason for the discrepancy in overall certainty between diagnosis and prognosis. We inferred that the QUADAS/QUADAS-2 scale used for diagnosis was more rigorous than the NOS scale used for prognosis study (most included cohort studies). However, considering the different stages of different study designs, we suggested the following framework of Whiting when conducting a quality assessment (60).

In addition, the meta-analysis conducted by Dai et al. provided three different pieces of evidence (high certainty evidence for DFS, moderate certainty evidence for OS and PFS). One possible explanation for the lower certainty evidence for OS and PFS is that they both have publication bias. Publication bias was an important factor leading to different quality levels measured by GRADE guidelines, and asymmetry of funnel plots was found in these two pieces of evidence from the same meta-analysis (44), which may arise from the low possibility of publication for studies with negative results. We graded the quality of evidence for the prognostic effect of HE4 only in LC as moderate (downgraded for inconsistency, *I*^2^ is 81%) The heterogeneity could be explained by ethnicity. Zhong et al. concluded that HE4 was associated with OS only in Asian populations but not in Caucasian patients (8).

### Explanation Findings From the Diagnostic Meta-Analysis

Our study found that HE4 played a potential role in the diagnosis of OC (20, 48, 52, 53) that was consistent with previous meta-analyses (9, 61). For example, the article reported a high pooled specificity (92%) for HE4, and the AUC of HE4 was higher than that of CA125, with values of 0.89 and 0.85, respectively (61). Besides, Ferraro et al. conducted a study and showed a similar result, and they found HE4 seems to be superior to CA125 in the terms of diagnostic performance (diagnostic sensitivity, specificity, positive likelihood ratios, and negative likelihood ratios) of patients with OC (20). There was sufficient evidence for HE4 is overexpressed in OC, which can reinforce the confidence of our result. The increased level of HE4 can help to improve the specificity for the diagnosis of OC. Dochez et al. reported a specificity of 90.4% for HE4, and the AUC was higher than CA125 alone, with the values of 0.91 and 0.83, respectively (62). Additionally, a study including 762 Korean patients showed that HE4 could be used for differentiating benign gynecological diseases and OC (9). The potential regulatory mechanism of HE4 in the cancer invasion and metastasis of OC could be attributed to the activation of MAPK and FOCAL signaling pathways (63). HE4 participates in the metastasis of OC by regulating the expression of extracellular matrix components, such as LAMC2 and LAMB3 (64).

Of all the certainties of evidence for the association, 50% was very low. We evaluated the quality of evidence on OC through GRADE, and most pieces of evidence were downgraded because of inconsistency (*I*^2^ > 50%), risk of bias (20, 48, 52), and publication bias (20, 48). Specifically, some meta-analyses had a high risk of bias for patient selection, index test (52), and reference standards (48). The difference between these two studies (48, 52) may be attributed to the number of publications (7 vs. 31) and sample type (urine vs. serum). Moreover, the methodological quality of six published meta-analyses by the AMSTAR was moderate, whereas for the remaining three meta-analyses, the methodological quality high. All these nine meta-analyses did not pay attention to the status of publication, such as searching gray literature, which included all languages or screening the references of the included articles. Considering that the gray literature in the search strategy and inclusion criteria can help to reduce the publication bias (65), we encouraged the authors of systematic analysis to search the gray literature.

In addition, the present umbrella review demonstrated that HE4 could play a probable role in the diagnosis of EC, and our results were in line with the previous systematic review and meta-analysis (21, 32). For example, the meta-analysis identified 12 articles dealing with HE4 and the diagnosis of EC and found that the sensitivity of 0.71 (95% *CI*: 0.56–0.82) and specificity of 0.87 (95% *CI*: 0.80–0.92) (21). Besides, one meta-analysis stated that HE4 is generally a better biomarker than CA125 in EC diagnosis by its higher sensitivity than CA125, and the author suggested that the combination of CA125 and HE4 may enhance the diagnostic sensitivity for patients with EC (43). Besides, the serum HE4 level in patients with EC was enhanced compared with that in benign patients (57, 66). The overexpression of HE4 was associated with cell proliferation, colony formation in soft agar, and the Matrigel invasion of EC cells (67). In addition, the level of HE4 was correlated with the depth of myometrial invasion, which was an important factor in the risk stratification or metastasis of EC (68).

Using GRADE guidelines, the six pieces of evidence from three meta-analyses were all downgraded by one level for inconsistency. The heterogeneity in most meta-analyses may be attributed to the population, intervention, controls, study design, threshold bias, and publication country (21, 50). Of note, we evaluated the indirectness using the test method for HE4 level [electrochemiluminescence (ECLIA) vs. ELISA] (69). Among the eight primary studies included in the meta-analysis (43), three studies used ELISA (26, 70, 71), whereas one study tested the HE4 level by ECLIA (72). Therefore, an indirectness of evidence existed. Moreover, the proportion of high methodological quality of meta-analyses evaluated by the AMSTAR was approximately 50% (3/6). All six meta-analyses did not show a study list of inclusion and exclusion, through which other scientists could repeat the study and confirm the authenticity of these results. Therefore, we encouraged the authors of meta-analyses to provide not only a list of included studies but also an exclusion study list.

We observed that the high expression of HE4 was associated with LC. However, the diagnostic effect of HE4 on LC has been limited. There were some results from other studies consistent with our conclusion (8, 42, 73). For example, an original study found that serum HE4 levels in patients with any histological type of LC (adenocarcinoma, squamous cell carcinoma, large cell carcinoma, and small cell lung carcinoma) were significantly increased compared with those in the control group (73). Thus, the overexpression of HE4 suggested a scientific basis for its potential diagnostic ability in LC. In addition, Cheng et al. conducted a meta-analysis of seven studies and found that the AUC was 0.856 for HE4 for patients with LC (42). In contrast, an original study found that the association between squamous cells LC was weak at 10% (74). The exact biological mechanisms of HE4 expression in LC were limited. One possible explanation was the effect of smoking, which may be correlated with lung chronic inflammation and HE4 levels (14, 75).

For diagnostic studies, the pieces of evidence (sensitivity and specificity) from a meta-analysis were both downgraded for the risk of bias (15). The methodological quality of most primary studies in the meta-analysis (15) was low using QUADAS-2, especially in the patient selection domain (5/21 were high risk, 3/21 were low risk with the remaining were unclear risk of bias). In the evaluation of LC by the AMSTAR, 75% of the meta-analyses exploring the function of HE4 in LC have high methodological quality. Remarkably, none of those meta-analyses display a CRD number that was registered on PROSPERO. Considering the advantages of prospective registration, such as study design, conduct procedure, and report results (76), we encouraged the author of meta-analyses to register before conducting a new project.

### Study Strengths

For strengths, to our knowledge, this is the first umbrella review to provide the most comprehensive critical appraisal of previously published systematic reviews and meta-analyses about the role of HE4 in the diagnosis and prognosis of diverse diseases. The methodological quality of the included systematic reviews and meta-analyses and the strength and credibility of associations were assessed by a unified method. At first, umbrella review is the review of existing systematic reviews and meta-analyses (27). Compared with an individual systematic review and/or meta-analysis, an umbrella review has a higher level of evidence, which can better promote clinical practice. Second, the quality of the included meta-analyses was moderate or high, which was evaluated by the AMSTAR tool. Articles in our umbrella review were partially or almost fully consistent with the standards of methodological quality, thereby suggesting the outcome of the role of HE4 in the diagnosis and prognosis of diseases with a high degree of credibility.

### Study Limitations

However, several caveats should be considered. For the present umbrella reviews, we only included the most recently published original studies, systematic reviews, and meta-analyses. First, meta-analyses published after our study and had yet to be assessed through meta-analyses will have an impact on our results. This limitation is shared by all umbrella reviews addressing clinical research. Second, the quality of this umbrella review depends on the quality of the articles included in the study. Some factors may have affected the strength and validity of evidence in meta-analyses, such as study design, sample size, and adjustment for confounders. For the original studies, the methods for assessing HE4 and the sample source were inconsistent, which might increase the risk of bias. However, we have assessed included meta-analyses by the AMSTAR tool to ensure their quality. After evaluation, the methodological quality was high or moderate. Third, the number of meta-analyses included in the umbrella review is limited, especially only four pieces of evidence from the prognostic meta-analysis. Given this limitation, we need to interpret the results of the present study with caution. Fourth, some studies failed to meet the requirements of the GRADE and cannot be evaluated. We did not reanalyze all the data, and the data were extracted from the original literature. Therefore, we encourage the remaining authors of the secondary study to comply with the protocol (28). Finally, most of the evidence from diagnosis and prognosis assessed by GRADE is moderate, low, and very low, and only one association was rated as high. It is important for us to interpret the results with caution. The low- and very low-quality evidence of the association between the role of HE4 in the diagnosis and the prognosis of diseases could only provide direction for future study.

## Conclusion

In summary, the high certainty evidence of HE4 was present for its prognostic role in cancers. HE4 could provide a suitable method for the diagnosis of diseases with moderate certainty evidence. We suggested that clinicians could use HE4 as a biomarker related to the diagnosis and prognosis of EC, OC, and LC in clinical work, so as to improve the understanding of disease diagnosis, efficacy, recurrence, outcome, and prognosis of patients. Considering that other diseases remain uncertain and inadequate, further studies are needed to better elucidate the diagnostic and prognostic role of HE4 in the future.

## Author Contributions

BL, Q-JW, and T-TG contributed to the study design. M-LS, Y-ZL, and X-YL collected the data and analyzed the data. M-LS, Z-YY, BL, Y-ZL, X-YL, F-HL, Y-FW, Z-YW, Q-JW, and T-TG wrote the first draft of the manuscript and edited the manuscript. All authors read and approved the final manuscript.

## Conflict of Interest

The authors declare that the research was conducted in the absence of any commercial or financial relationships that could be construed as a potential conflict of interest.

## Publisher’s Note

All claims expressed in this article are solely those of the authors and do not necessarily represent those of their affiliated organizations, or those of the publisher, the editors and the reviewers. Any product that may be evaluated in this article, or claim that may be made by its manufacturer, is not guaranteed or endorsed by the publisher.
